# Improved catalytic combustion of methane using CuO nanobelts with predominantly (001) surfaces

**DOI:** 10.3762/bjnano.9.235

**Published:** 2018-09-24

**Authors:** Qingquan Kong, Yichun Yin, Bing Xue, Yonggang Jin, Wei Feng, Zhi-Gang Chen, Shi Su, Chenghua Sun

**Affiliations:** 1 School of Mechanical Engineering, Chengdu University, Chengdu 610106, Sichuan, PR China; 2 School of Chemistry, Faculty of Science, Monash University, VIC 3800 Australia; 3 CSIRO Energy, PO Box 883, Kenmore, QLD 4069 Australia; 4 School of Environment and Resources, Southwest University of Science and Technology, Mianyang 621000, PR China; 5 CSIRO Energy, 1 Technology Court, Pullenvale QLD 4069, Australia; 6 Centre for Future Materials, University of Southern Queensland, Springfield QLD 4300, Australia; 7 Department of Chemistry and Biotechnology, Faculty of Science, Engineering & Technology, Swinburne University of Technology, Hawthorn, VIC 3122, Australia

**Keywords:** catalytic oxidation, copper oxide, density functional theory, methane

## Abstract

Through computational calculations, CuO(001) has been identified as an active surface for methane oxidation. Experimental validation with CuO nanobelts comprised of predominantly (001) surfaces has been performed and it is confirmed that the performance of such nanobelts is much higher than normal nanoparticles and nanowires. First principle calculations further clarified that two-coordinated oxygen plays a key role for methane adsorption and oxidation.

## Introduction

Methane (CH_4_), as the main component of natural gas, offers significant environmental advantages over conventional gasoline and diesel [1–3]. However, its thermal combustion is often at a flame temperature up to ≈2,000 K [4], which favours the formation of nitrogen oxide (NO*_x_*) pollutants. To suppress such reactions, a lower flame temperature is targeted, but this potentially results in incomplete combustion with large CO and unburned hydrocarbon (UHC) emissions [5], which underlines the need to develop high-performance catalysts for CH_4_ oxidation at low temperature. Except as fuel in natural gas, much of the CH_4_ that is released is from industrial applications, such as ventilation air methane (VAM) from underground coal mining. This is a serious issue because CH_4_ has severe global warming effects, around 25 times greater than that of carbon dioxide (CO_2_). For a more efficient use of natural gas and to minimize the direct emission of CH_4_, the catalytic oxidation of CH_4_ at low temperature has been investigated extensively over the last decades [5–8].

Noble metals have been reported as effective catalysts for the complete oxidation of methane [2,5,9–17], particularly supported Pd nanoparticles [15–17], while the high price and poor thermal stability limit their large scale application. To address this issue, low-cost alternatives, such as transition metal (TM) oxides and various complex structures (e.g., perovskite, spinel and hexaaluminate) have been tested as catalysts for CH_4_ oxidation. But so far their performance is still much lower than noble metals. An ideal catalyst for CH_4_ oxidation should have a high capacity to adsorb CH_4_ (particularly important for CH_4_ oxidation at low concentration, for example, VAM mitigation), activate the C–H bond and split the O_2_ molecule. However, exposed catalyst surfaces are often highly stable and thus can hardly satisfy all of the above criteria, which explains the poor performance of most metal oxides.

To improve the reactivity of metal oxides, surfaces with high energy (and thus low surface area), namely minority surfaces, may offer new opportunities. Although minority surfaces often diminish quickly during crystal growth due to their low stability, they can be stabilized by controlling the synthesis conditions, as previously demonstrated in the literature [18–20]. In terms of reactivity, minority surfaces have a large amount of lowly coordinated atoms that can be actively involved in surface reactions, including adsorbing CH_4_ and stabilizing intermediates [7]. Following this strategy, this work explores the catalysis of copper oxide (CuO), a promising catalyst for CH_4_ oxidation as identified in the literature [8]. Different from these reports, we focus on the performance of the minority surface (001).

## Results and Discussion

Starting with computational calculations, CuO surfaces with low indexes (including (110), (111), (101), (010), (011) and (001)) have been systematically screened under the scheme of density functional theory. The stability of these surfaces has been reported earlier as (010) > (011) > (100) > (101) > (110) > (001) [21], according to which 001 is the most reactive surface, which is consistent with our work as demonstrated below.

**Figure 1:**
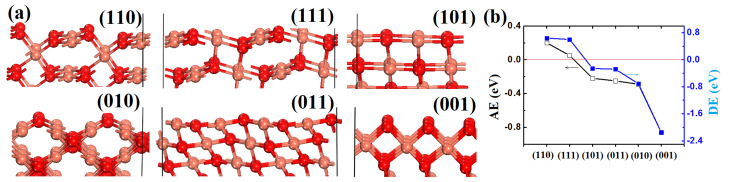
Computational screening. (a) Models for six low-index surfaces (unrelaxed); (b) Calculated values of AE and DE. Cu and O are shown as rose-carmine and red, respectively.

Two indicators, namely the adsorption energy (AE) and dissociation energy (DE, energy change from physical adsorption to dissociative adsorption), are employed for the analysis. The surface models are shown in Figure 1a. Given that the catalysts are exposed to abundant oxygen, O or O/Cu-mixed termination is employed in the models. Among them, five are featured with mixed O/Cu surface atoms, except (001) which is fully covered by two-coordinated oxygen (O_2c_) before it is relaxed. Very recently, O_2c_ has been identified as an active site for CH_4_ oxidation in the case of Co_3_O_4_ whereby CuO(001) offers an ideal opportunity to test this hypothesis [22].

The AE and DE are calculated and presented in Figure 1b, with negative AE (DE) indicating that the adsorption (dissociation) is favorable. Based on the calculated AE, it is found that the physical adsorption of CH_4_ on (101), (011), (010) and (001) can occur without additional energy input, with an AE between −0.22 and −0.86 eV. The (001) surface, with AE = −0.86 eV, has been identified as the surface that offers the strongest adsorption capacity among all these surfaces. For CH_4_ oxidation, the dissociation to form CH_3_ and H is critical and difficult because strong C–H bonds need to be broken; therefore, the calculated DE is indicative of the oxidation difficulty. Interestingly, the (001) surface has been identified again as the most reactive surface for CH_4_ dissociation, with DE = −2.16 eV, which is much higher than that of the other surfaces. On the basis of the above computational screening, (001) is the optimal surface for CH_4_ oxidation among these tested surfaces. From the analysis of the surface, this is not surprising because (001) is strongly polarized and shows high reactivity due to the high ratio of lowly coordinated oxygen – a feature that has been employed for gas sensing and Li-ion batteries [21].

**Figure 2:**
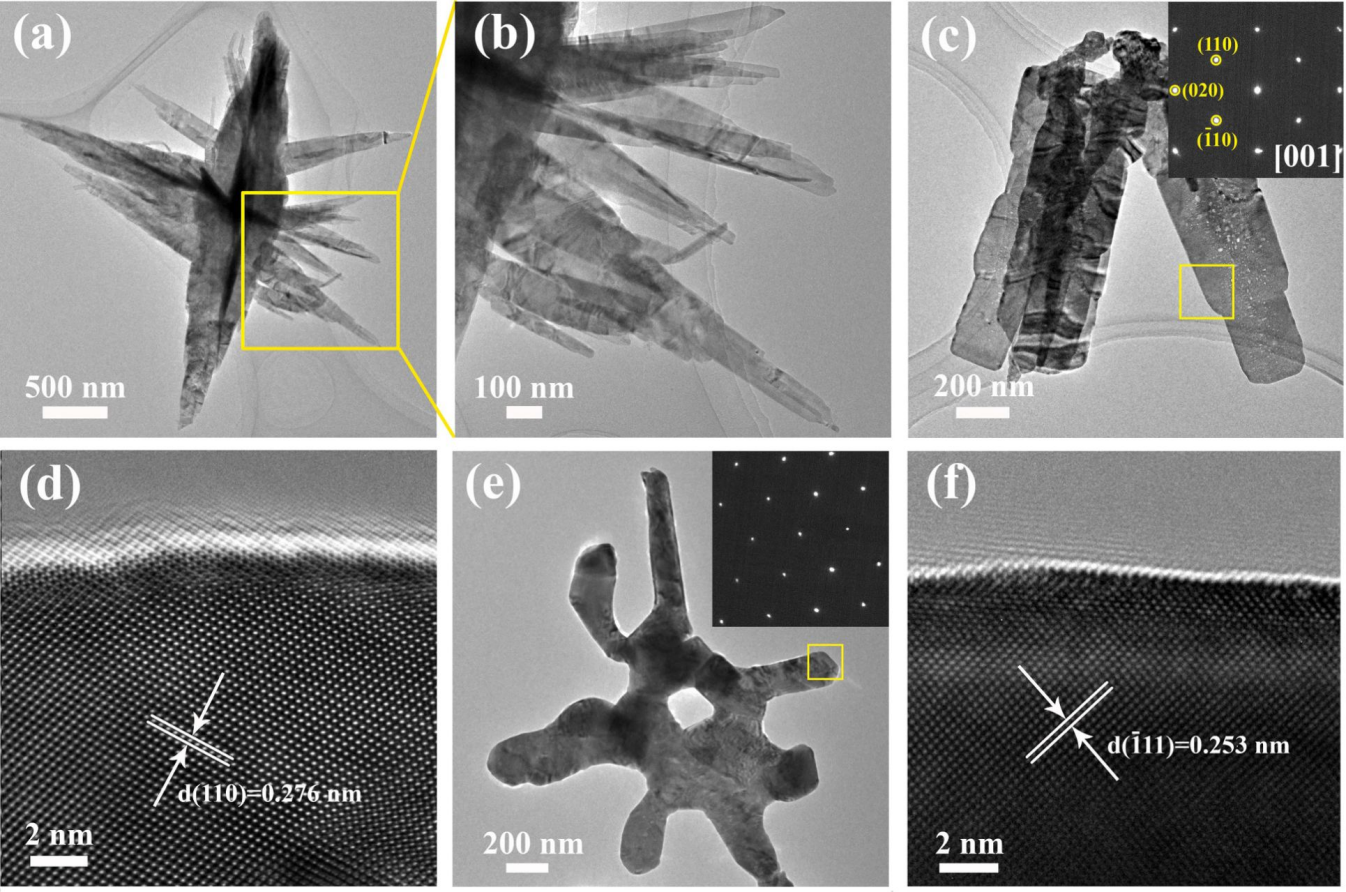
CuO catalyst characterization. (a,b) TEM images of CuO NWs; (c) TEM image of CuO NBs (inset is the SAED pattern of the selected yellow square area); (d) HRTEM along the [1] direction; (e,f) TEM images of a CuO nanobelt after catalysis tests at 650 °C.

Now we turn to the experimental validation, starting with the synthesis of CuO nanowires (NWs) and CuO nanobelts (NBs) comprised of predominantly (001) surfaces, which is performed based on the method reported by Van Tendeloo et al. [23]. For the NWs and NBs synthesized in this work, a high percentage of the surface area can be attributed to (001) surfaces, particularly for the NBs whose (001) surfaces are the predominant surface found, as examined by transmission electron microscopy (TEM). Figure 2a,b shows the NWs which have a diameter of 20–100 nm and length of 1–3 μm, and Figure 2c shows the thin NBs, with a width of 200–300 nm and a length of ≈1 μm. The selected area electron diffraction patterns (SAED), projected from the [1] zone axis of the yellow square area (see the inset in Figure 2c), reveals the monoclinic structure and that the diffraction spots are ascribed to the (110), (−110), and (020) planes. High-resolution TEM images, as shown in Figure 2d, confirm that the exposed surface of the NBs is the (001) facet and the edge is (100). As shown in an early study [21,23], CuO NWs or NBs having predominantly (001) surfaces can be obtained when Cu(OH)_2_ is employed as a nanowire precursor for decomposition. The mechanism of shape-reserved transformation from Cu(OH)_2_ to CuO has been previously discussed in the literature [24–26].

Prior to the performance tests, the crystal structure of the collected samples was further examined by X-ray diffraction with commercial CuO nanoparticles (NPs, Aldrich 450812) as a reference for comparison. The NP, NW and NB samples show the similar profiles, confirming that they have the same phase (CuO, space group: C2/c2(15), *a* = 4.685 Å, *b* = 3.425 Å, *c* = 5.130 Å), as shown in Figure S1, Supporting Information File 1. However, the NB sample shows a much stronger (002) peak, suggesting a preferred (001) termination. It is also found that the NP, NW and NB samples have a similar surface area of around 1 m^2^/g as measured by the Brunauer–Emmett–Teller (BET) method from N_2_ adsorption isotherms at 77 K, suggesting that the performance difference presented below does not come from the difference of the surface area.

**Figure 3:**
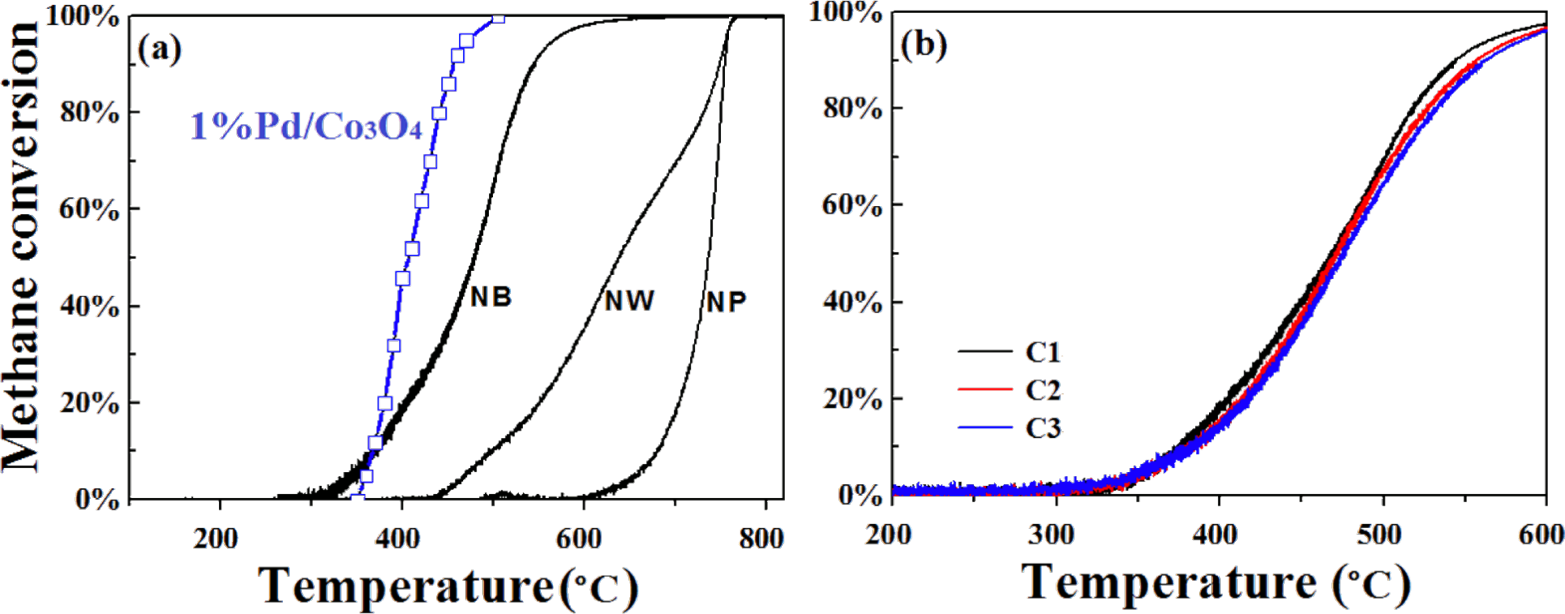
CH_4_ conversion against the temperature. (a) Heating profile (*T*_max_ = 850 °C) for NBs, NWs and NPs, with 1% Pd/Co_3_O_4_ as a reference. (b) Heating profile (*T*_max_ = 600 °C) for NBs tested for three cycles, labelled as C1, C2 and C3.

We now compare the catalytic performance of different CuO catalysts for CH_4_ oxidation. We began with catalytic oxidation tests, starting from 150 °C up to the maximum testing temperature, *T*_max_ = 850 °C. After the first cycle of the test (denoted as C1), the catalytic bed was cooled down from 850 to 150 °C followed by the second test cycle (C2). Figure 3a shows the C1-performance of the NP, NW and NB samples where the oxidation activity follows the order: NBs > NWs > NPs. It is particularly notable that the NB performance is even close to that of 1% Pd loaded on Co_3_O_4_ [27], which itself shows excellent catalytic capacity. Supermolecular Pd@CeO_2_ core–shell structures can offer exceptionally high performance for methane oxidation, achieving complete conversion even below 400 °C [6]. Different from Pd catalysts, CuO NBs, as a low-cost metal oxide material, do not have to be loaded on a support and do not use additional dispersion agents, and are thus a more effective catalyst per volume and weight when assembled on the reactor.

The aggregation of catalyst nanoparticles upon high-temperature calcination is a typical issue for CH_4_ oxidation, which was also observed in our experiments. All three CuO catalysts demonstrated poor thermal stability leading to significantly deteriorated catalytic performance in C2 compared to that in C1 when the combustion temperature was as high as 850 °C. Therefore, CH_4_ oxidation catalyzed by NBs at lower temperature (600 °C) was tested, and the results are shown in Figure 3b. These results show that the catalytic performance can be kept stable after three circles, achieving *T*_10_ = 375–378 °C, *T*_50_ = 468–475 °C, *T*_90_ = 544–562 °C and around 97% of CH_4_ conversion at 600 °C, indicating that the belt shape may be helpful to suppress catalyst aggregation. However, the stability is still poor as the nanobelts tend to aggregate when high temperature treatment is employed (650 °C) as shown in Figure 2e. Moreover, the surface layers show higher crystallization after catalysis tests, as shown in Figure 2f, indicating that additional efforts to improve the thermal stability of these nanostructures is needed.

Although the calculated AE and DE can provide an explanation for the effect of morphology on catalysis performance, we further investigated the mechanism of full CH_4_ oxidation over CuO(001), which is informative for the further improvement and the design of new catalysts. Generally, Cu does not show strong magnetization in its neutral or fully oxidized forms, but spin-polarization for lowly coordinated Cu on the surface and radicals involved in CH_4_ oxidation deserves serious consideration. For instance, the difference in calculated adsorption energies with and without spin-polarization can be as high as 0.1–0.2 eV, with geometries showing slight differences too. Therefore, all energies and geometries shown below are based on spin-polarized calculations.

**Figure 4:**
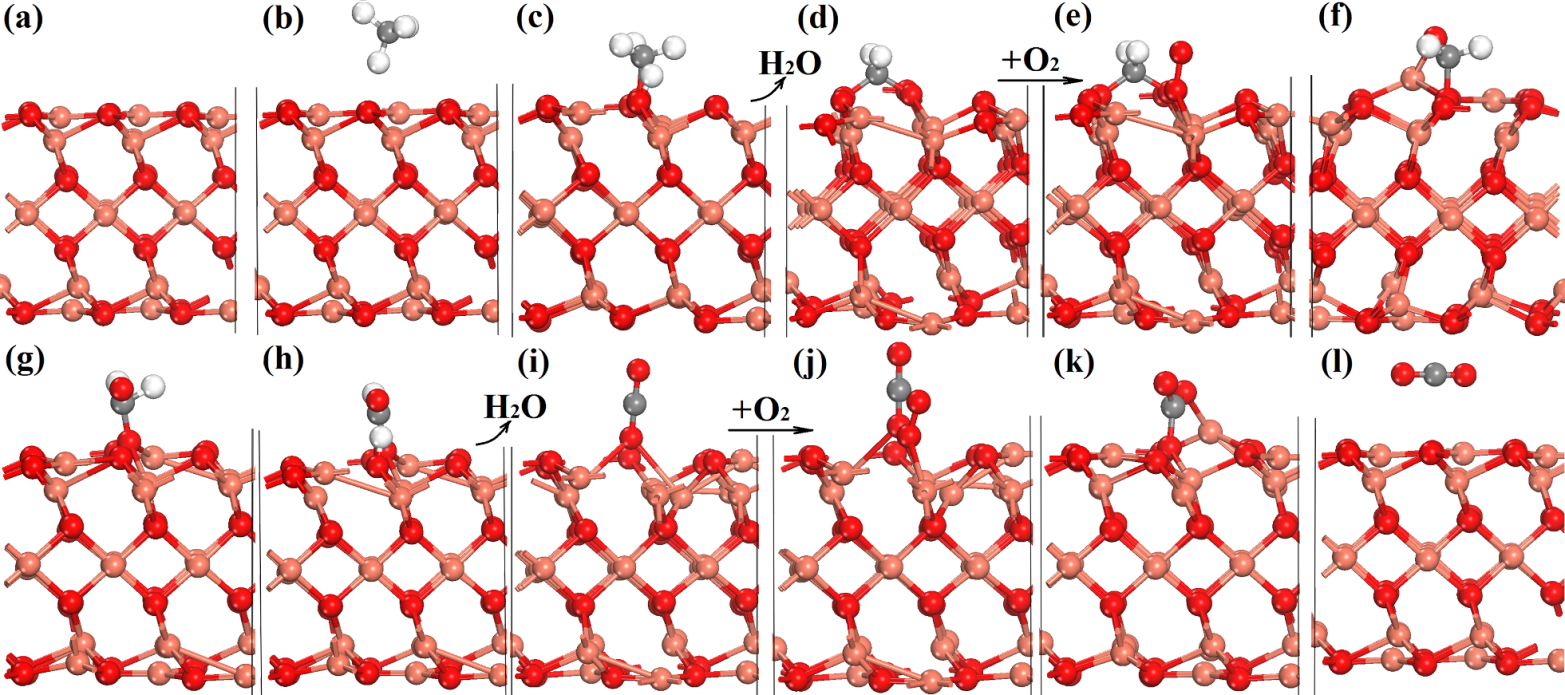
CH_4_ oxidation mechanism by computational calculations. (a) Clean (001); (b) CH_4_ physical adsorption; (c) CH_4_ dissociative adsorption; (d) CH_2_* with oxygen vacancy (OV) presented; (e) CH_2_* with O_2_ adsorbed; (f) CH_2_* rotating and interacting with O_2_*; (g) transition state (TS) state COH_2_*; (h) CHO* + H*; (i) CO* with OV presented after releasing one H_2_O; (j) CO* with O_2_ adsorbed; (k) TS for O transfer to CO*; (l) CO_2_ physical adsorbed. Cu, O, C and H are shown as rose-carmine, red, grey and white spheres, respectively.

Starting from clean (001), CH_4_ is firstly adsorbed with AE = −0.86 eV, followed by a spontaneous dissociation with CH_3_ and H adsorbed on surface oxygen, as depicted in Figure 4a–c. Similar as discussed in an early publication [28], surface oxygen is often actively involved and may cleave H from CH*_x_* to form –OH and H_2_O, generating an oxygen vacancy (OV), which can be filled with reactant O_2_, leading to its dissociation and further oxidizing CH*_x_* intermediates, as shown in Figure 4d–f. With oxygen transferring to bond with carbon, the hydrogen in CH*_x_* can shift to surface oxygen again with an energy release of 3.66 eV and 1.46 eV, indicating that such a shift is highly favorable, as shown in Figure 4g–i. Again, an OV is generated and refilled by O_2_ when H_2_O is released, and similarly O_2_ is dissociated to release atomic oxygen after exceeding the small barrier of 0.47 eV, which can oxidize adsorbed CO to form CO_2_, as outlined in Figure 4i–l.

**Figure 5:**
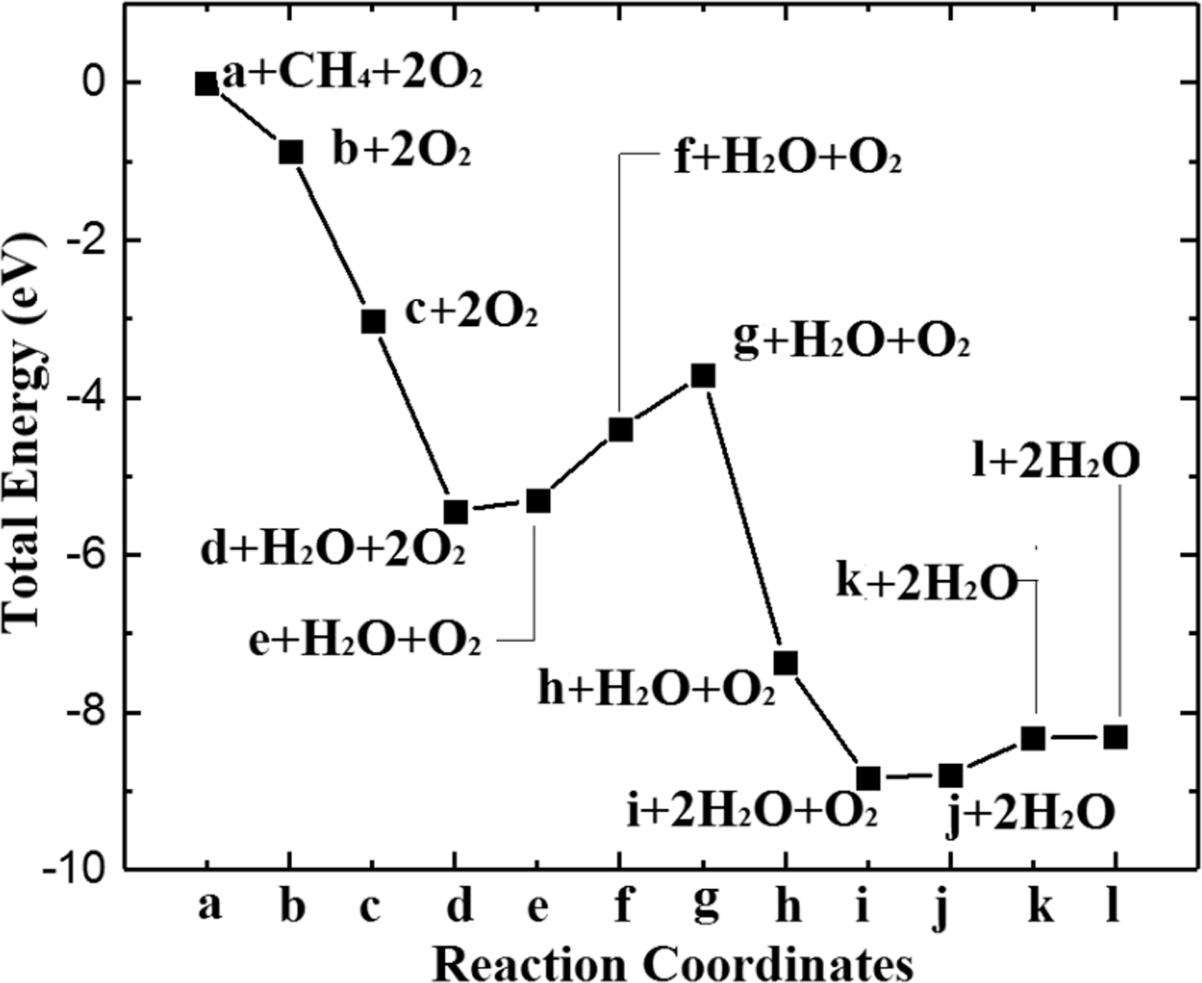
Energy profile for CH_4_ oxidation. The geometries are shown in Figure 4.

The energy profile for the above full process is given in Figure 5 in units of eV, using the total energy of a clean (001) surface and free gas-phase CH_4_ and O_2_ as the reference. According to the results, the early oxidation to release the first H_2_O (oxygen is from the surface) is exothermic and generates an OV (see Figure 4c). This is followed by O_2_ dissociation which occurs over the vacancy with a maximum energy barrier of 0.91 eV. This is informative because it indicates that the key role of OV is to activate O_2_ and release a highly active oxygen atom, although a barrier of 0.91 eV is found for O_2_ dissociation in the case of CuO(001), but the dehydrogenation from CH*_x_* becomes exothermic, confirming the importance of surface oxygen and oxygen vacancies, as suggested by Hu et al. [22] and Jin et al. [28]. According to this understanding, the catalysis performance may be further improved by adding alloy elements or surface doping to reduce the barrier.

## Conclusion

In summary, Cu(001) was identified as being the most promising among six low-index surfaces tested for CH_4_ oxidation based on the criteria of adsorption energy and dissociation energy. These calculations were experimentally examined with the use of CuO NBs comprised of predominantly (001) surfaces, which performed better than CuO NBs and NPs, achieving almost complete oxidation at a temperature of 600 °C. CuO NBs, employed as low-cost catalysts, can be used without additional agents to disperse or support them. They can therefore be loaded with higher concentration in limited space or weight applications, making CuO NBs a promising replacement to expensive Pd-based catalysts. Additional efforts are needed to improve the thermal stability of such nanostructures as they tend to aggregate after high temperature treatment.

## Experimental

### Synthesis of CuO nanowires and nanobelts

For a typical sample, 1 g of Cu(NO_3_)_2_ was dissolved in 100 mL distilled water and 30 mL ammonia solution (NH_3_·H_2_O, 0.15 mol/L) was added under vigorous stirring. Then, NaOH solution (1 mol/L) was added dropwise to the above solution and the pH value was adjusted to 9–10. The resulting blue Cu(OH)_2_ precipitate was filtered and washed several times with distilled water to obtain a solid product. CuO NWs were prepared by heating one part of the as-obtained solid product at 130 °C for 10 h in an oven. The other part was dissolved in 50 mL distilled water, then transferred to an autoclave and hydrothermally treated at 130 °C for 10 h. The black solid product was washed with deionized water and dried at 60 °C overnight to get CuO NBs [23].

### Methane catalytic oxidation test

Methane catalytic oxidation experiments were carried out in a fixed-bed reactor (U-shaped quartz tube) with an inner diameter of 6 mm at atmospheric pressure. 35 mg of the catalysts was loaded into the reactor and supported by quartz wool. A mixed reaction gas containing 0.6 vol % CH_4_, 19.6 vol % O_2_, 0.15% CO_2_ and balance N_2_ was flowed through the catalyst bed at a flow rate of 20 mL min^−1^, corresponding to a gas hourly space velocity of 34,000 mL h^−1^ g^−1^. Prior to the catalytic tests, the catalyst was pretreated under the reaction atmosphere for 60 min at 250 °C and then cooled down to 150 °C. Once the temperature was stable at 150 °C, CH_4_ oxidation catalytic performance was evaluated by heating the catalyst bed at 10 °C min^−1^ to 850 or 600 °C. The composition of the effluent gases were monitored on-line using a quadrupole mass spectrometer (Hiden MS HPR20) with a secondary electron multiplier detector. Methane conversion was defined as: (influent concentration of CH_4_ − effluent concentration of CH_4_)/influent concentration of CH_4_ × 100%.

### Theoretical calculations

Spin-polarized DFT calculations were performed under the generalized gradient approximation (GGA) [29] together with the functional by Perdew, Burke, and Ernzerhof [30], which was embedded in the Vienna Ab-Initio Simulation Package [31]. The calculation was carried out with a kinetic cutoff energy of 380 eV, with the use of the projector augmented wave (PAW) method. The k-space was sampled by the gamma point based on our tests. The k-point and cutoff energy were tested with higher computational settings, as 3 × 3 × 1 and 450 eV, leading to the difference of less than 0.1 eV in terms of the adsorption energies. The van der Waals (vdW) interaction was considered with the use of the DFT-D3 method, as developed by Grimme and Jónsson [32–33]. The CuO surfaces were modelled by 2 × 2 supercells, shown as slabs with thicknesses in the range of 10–14 Å, depending on the surface orientation, and a vacuum space of 12 Å was applied over the surface. The transition state (TS) was identified through the dimer method [33], for which the searching is carried out by a series of intermediate states generated among the reaction path towards the formation of the product and further optimized with one static degree of freedom (in our case, one bond length associated with the change from reactant to product). Based on our tests, both spin-polarization and vdW corrections are necessary, especially for the calculation of intermediate radicals involved in CH_4_ oxidation (e.g., CH_2_*, CH* and O*).

During the quick screening of surfaces for CH_4_ adsorption, the adsorption energy (AE) and dissociation energy (DE) from physical adsorption to dissociative adsorption have been employed, which are defined as follows.

AE = *E*(CH_4_*) – *E*(surface) – *E*(CH_4_-gas phase)

DE = *E*(CH_3_*-H*) – *E*(CH_4_*)

To understand the catalysis mechanism, full methane oxidation, CH_4_ + 2O_2_ → CO_2_ + H_2_O, has been investigated in which the total energy of CH_4_, CO_2_ and H_2_O was calculated as −24.02 eV, −23.64 eV and −14.23 eV, respectively. The total energy of O_2_ was derived from H_2_O and H_2_, giving −9.97 eV. Based on the above, the reaction energy for CH_4_ oxidation was calculated as −8.14 eV, which is close to the experimental data (Δ*H* = −8.31 eV at room temperature).

## Supporting Information

File 1 X-ray diffraction profile.
