# Supplementary material for: Improved catalytic combustion of methane using CuO nanobelts with predominantly (001) surfaces
Source: Beilstein J Nanotechnol. 2018 Sep 24;9:2526–32. doi: 10.3762/bjnano.9.235 (PMC6176823; doi:10.3762/bjnano.9.235)
Supplement: File 1 — X-ray diffraction profile. [file Beilstein_J_Nanotechnol-09-2526-s001.pdf]

Supporting Information

for

Improved catalytic combustion of methane using CuO nanobelts with predominantly (001) surfaces

Qingquan Kong[1], Yichun Yin[2], Bing Xue[3,4], Yonggang Jin*[5], Wei Feng[1], Zhi-Gang Chen[6], Shi Su[5] and Chenghua Sun*[7]

Address: [1]School of Mechanical Engineering, Chengdu University, Chengdu 610106, Sichuan, PR China, [2]School of Chemistry, Faculty of Science, Monash University, VIC 3800 Australia, [3]CSIRO Energy, PO Box 883, Kenmore, QLD 4069 Australia, [4]School of Environment and Resources, Southwest University of Science and Technology, Mianyang 621000, PR China, [5]CSIRO Energy, 1 Technology Court, Pullenvale QLD 4069, Australia, [6]Centre for Future Materials, University of Southern Queensland, Springfield QLD 4300, Australia and [7]Department of Chemistry and Biotechnology, Faculty of Science, Engineering & Technology, Swinburne University of Technology, Hawthorn, VIC 3122, Australia

Email: Yonggang Jin - yonggang.jin@csiro.au; Chenghua Sun - chenghuasun@swin.edu.au

*Corresponding author

X-ray diffraction profile

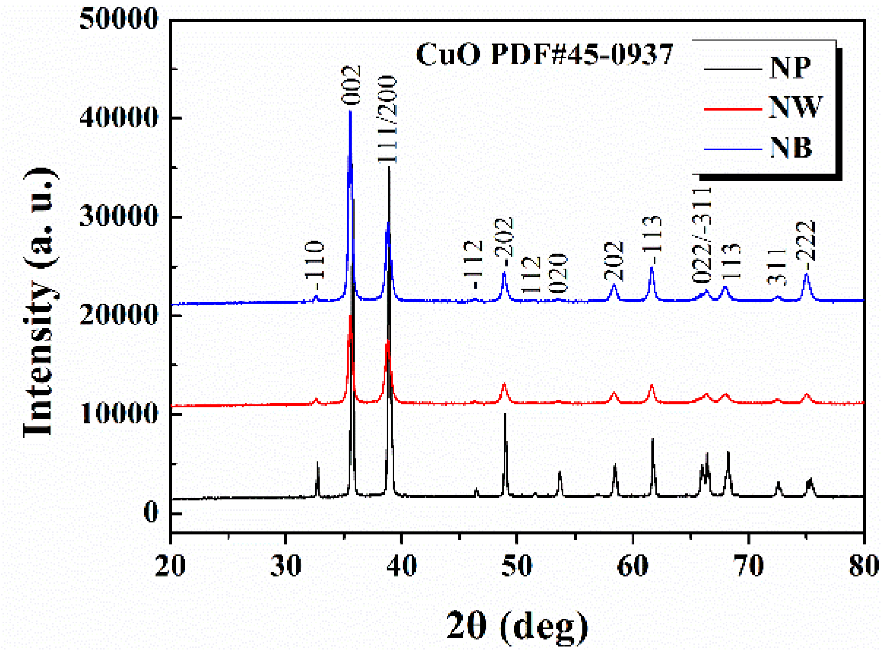

Figure S1: X-ray diffraction profile for CuO nanoparticles (NP), nanowires (NW) and nanobelts (NB).
